# Immunosenescence, Immune Fitness and Vaccination Schedule in the Adult Respiratory Patient

**DOI:** 10.1016/j.opresp.2022.100181

**Published:** 2022-05-16

**Authors:** Felipe Villar-Álvarez, David de la Rosa-Carrillo, Fernando Fariñas-Guerrero, Carlos A. Jiménez-Ruiz

**Affiliations:** a Pneumology Department, IIS Fundación Jiménez Díaz, CIBERES, Universidad Autónoma of Madrid, Madrid, Spain; b Pneumology Department, Hospital de la Santa Creu i Sant Pau, Barcelona, Spain; c Institute of Clinical Immunology and Infectious Diseases, Ynmun Group, ZEIG (Zoonotic and Emerging Infections Group), GEVIG (Study Group on Immunity, Infection and Vaccination of Immunocompromised and Geriatric Patients), Medical Microbiology Department, Faculty of Medicine, University of Malaga, Malaga, Spain; d Specialised Smoking Unit, Hospital Clínico San Carlos, Madrid, Spain

**Keywords:** Immunosenescence, Immune fitness, Vaccine, Vaccination, COVID-19, Influenza, Pneumococcus, Whooping cough, Herpes zoster, Immunosenescencia, Fitness inmunológico, Vacuna, Vacunación, COVID-19, Gripe, Neumococo, Tosferina, Herpes zóster

## Abstract

Immunosenescence is the gradual deterioration of the immune system caused by advancing age. It is associated with a reduced ability to respond to infections and develop long-term immune memory. It plays a key role in the development of respiratory diseases that are more common in older people, such as asthma, COPD, diffuse interstitial disease and respiratory infections in the elderly.

We call immune fitness the establishment of lifestyle habits that can improve our immune capacity. We now know that good eating habits, good social relationships, not smoking, limiting alcohol consumption, exercising, controlling stress levels and establishing a proper vaccination programme can slow down the process of immunosenescence.

Influenza and pneumococcal vaccines (PCV13 and PPSV23 conjugate) are well established in the adult vaccination schedule. The new pneumococcal vaccines PCV15 and PCV20 will help to extend protection against pneumococcal disease in adults. The vaccine against COVID-19 is currently the most useful tool to prevent the disease and reduce its pathogenicity. COPD patients and others with respiratory diseases may benefit from prevention of herpes zoster and *Bordetella pertussis* through vaccination. Respiratory syncytial virus (RSV) vaccine may be another vaccine to be added to the schedule, pending the results of its studies.

## Abstract (es)

La inmunosenescencia es el deterioro gradual del sistema inmune provocado por el avance de la edad. Se asocia a una menor capacidad para responder a las infecciones y desarrollar memoria inmune a largo plazo. Es parte fundamental en el desarrollo de las enfermedades respiratorias más frecuentes en edades avanzadas, como el asma, la EPOC, la patología intersticial difusa y las infecciones respiratorias del anciano.

Llamamos fitness inmunológico al establecimiento de unos hábitos de vida que puedan mejorar nuestra capacidad inmunitaria. Actualmente sabemos que tener buenos hábitos alimentarios, buenas relaciones sociales, no fumar, limitar el consumo de alcohol, hacer ejercicio, controlar los niveles de estrés y establecer un correcto programa de vacunación permiten ralentizar el proceso de inmunosenescencia.

Las vacunas de la gripe y las antineumocócicas (la conjugada PCV13 y la PPSV23) están bien establecidas en el calendario vacunal del adulto. Las nuevas vacunas antineumocócicas PCV15 y PCV20 van a servir para ampliar la protección contra la enfermedad neumocócica en el adulto. La vacuna contra la COVID-19 es, en el momento actual, la herramienta más útil para prevenir la enfermedad y disminuir su patogenicidad. Los pacientes con EPOC y otros con enfermedades respiratorias podrían beneficiarse de la prevención del herpes zóster y *Bordetella pertussis* mediante la vacunación. La vacuna contra el virus respiratorio sincitial (VRS) puede ser otra de las siguientes que formen parte de este calendario, en espera de los resultados de sus estudios.

## Introduction

The COVID-19 pandemic has served to raise awareness not only among the general population but also among health professionals of the importance of vaccination as a preventive measure against a number of infectious diseases. But if this is true for many pathologies, it is even more evident in infectious diseases whose target organ is the lung.^1^

This pandemic has also raised awareness of two concepts, immunosenescence, as part of the ageing of our immune system, and immune fitness, with the aim of training it, improving it and delaying its senescence.^2^ The former is a fundamental part of the development of respiratory diseases and their infectious complications. The latter could prevent them and improve the quality of life of these patients. Both will constitute an important part of the management of patients with chronic and acute respiratory pathologies.

Vaccines have historically been a powerful tool for combating infectious diseases and the pandemics they cause, and one of the most effective public health measures. Vaccines have evolved from the first vaccines composed of live micro-organisms to conjugate vaccines and the most current vaccines made from genetically engineered microbial material. The airways are a common source of infection and even more so in patients with chronic respiratory disease. The vaccination schedule for children is well established, but is lacking in adults, especially in chronic respiratory patients.

We will now address the most recent knowledge on immunosenescence in patients with respiratory pathology, immune fitness and its key aspects in order to advise these patients on how to improve their immune response, and what recommendations might be established in the vaccination schedule of the adult respiratory patient.

## Immunosenescence in the patient with pulmonary pathology

### Basic concepts

Ageing is described as a set of biological, cellular, sub-cellular and molecular changes that progressively develop with advancing age. These changes impair organ function and increase body frailty, favouring the development and progression of many diseases. Considering that the population over 65 years of age has progressively increased over the last decades and is expected to increase even more, it seems clear that ageing has and will have a significant social and public health impact on a global level.

**Fig. 1:**
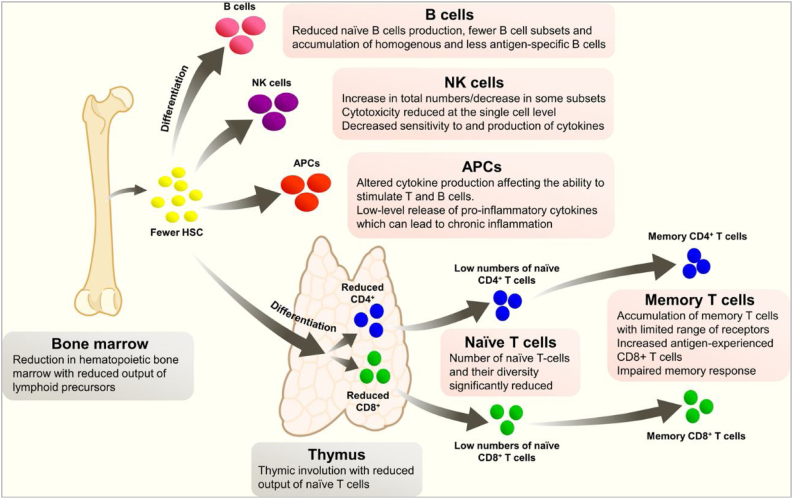
Changes in the immune system with age.

Reproduced from: Laupèze B, et al.^3^ Creative Commons Attribution 4.0 International License (https://creativecommons.org/licenses/by/4.0/).

Immunosenescence is the gradual deterioration of the immune system caused by advancing age. It is associated with a reduced ability to respond to infections and develop long-term immune memory. It is not a random phenomenon, as it seems to inversely repeat an evolutionary pattern, and most of the affected parameters seem to be under genetic control. Immunological changes in ageing include a reduction in hemopoietic bone marrow and thymic involution, which reduces the production of various lymphoid precursors and their subsequent maturation (Fig. 1).^3^ These changes lead to a poorer response to aggression, but also to a state of chronic inflammation, due to an increased release of pro-inflammatory cytokines.

A phenomenon closely related to immunosenescence is inflammaging, a term that describes the inflammatory state characteristic of ageing, which is due to chronic exposure to endogenous and exogenous antigens, as well as stress.^4^ The response to these exposures results in a Th2-dominant pro-inflammatory state that promotes molecular, cellular, organ and systemic damage, and over time decreases the ability to respond to new antigens. There is therefore a bidirectional relationship between immunosenescence and inflammation: immune dysfunction leads to chronic inflammation, and *inflammaging* impairs the immune response.

**Fig. 2:**
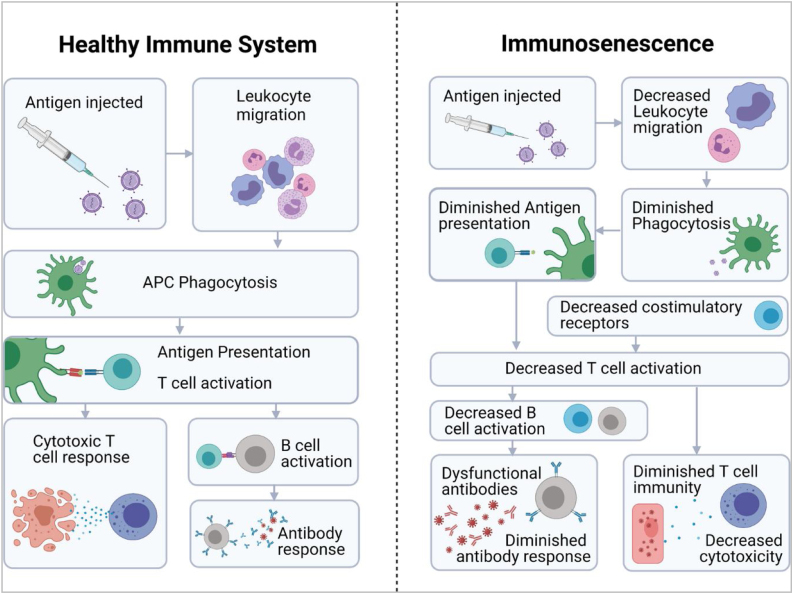
Effect of immunosenescence on the immune response to vaccines.

Reproduced from: Maturitas, Vol 147, Jack Feehan, Nicholas Tripodi, Vasso Apostolopoulos, *The twilight of the immune system: The impact of immunosenescence in aging*, 7–13, 2021, (6) with permission from Elsevier.

All these phenomena ultimately mean that patients have an “immunological age” that is higher than their chronological age,^5^ which is associated with an increased incidence of tumours or autoimmune/inflammatory diseases, increased susceptibility to infections and a lower response to vaccination.^6^, ^7^ A lower immune response to a variety of vaccines has been demonstrated in the elderly population, including diphtheria, pertussis, herpes zoster, pneumococcal polysaccharide and trivalent influenza vaccines (Fig. 2).^6^ In the case of SARS-CoV-2 vaccine, there is evidence of a more rapid decline in neutralising antibody titre in the elderly population than in the young.^7^

### Immunosenescence and respiratory diseases

The factors that most influence lung ageing are genetic predisposition, systemic exposure (such as changes in gut microbiota or systemic diseases) and environmental exposure (both particulate and microbial).^8^ However, immunosenescence is a key part in the development of some respiratory diseases that are more common in older age, such as adult asthma, chronic obstructive pulmonary disease (COPD), diffuse interstitial disease and respiratory infections in the elderly.^2^

Unlike asthma in children and young people, the pathophysiology of late-onset asthma is less well established. Factors such as atopy/allergy are less influential and appear to be related to viral infections or irritants, which in a context of immunosenescence lead to persistent inflammatory changes, mainly of a Th2 and neutrophilic type.^8^ Immunosenescence also affects patients with long-standing asthma, altering their basal inflammation and favouring a chronic inflammation with distinctive characteristics, which favours airway remodelling. Lastly, it also conditions asthmatic exacerbations in the elderly, due to the worse clearance of pathogens and a greater inflammatory response against them.^9^

**Fig. 3:**
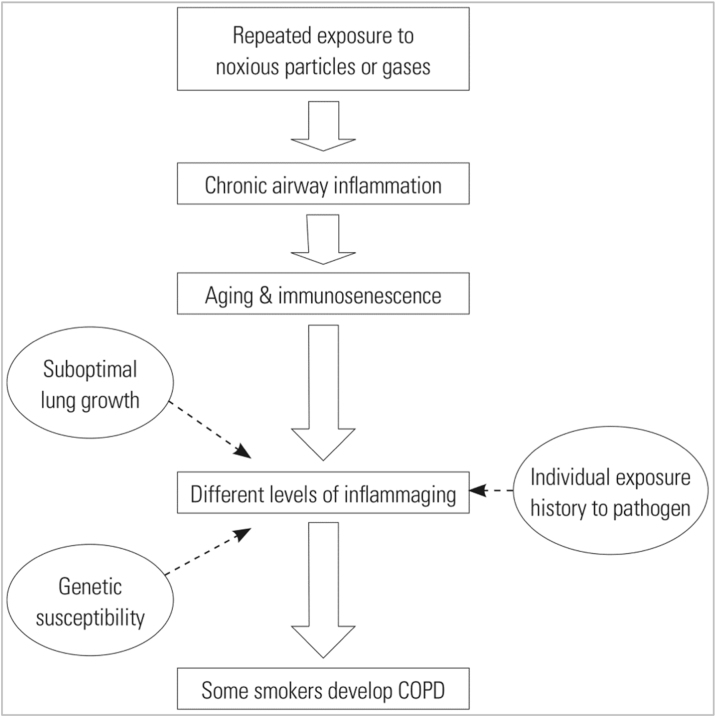
Overview of immune hypotheses on COPD. The different levels of inflammation caused by immunosenescence determine the susceptibility of individuals to COPD.

Reproduced from: Cho WK, Lee CG, Kim LK. *COPD as a Disease of Immunosenescence*. Yonsei Med J. 2019 May;60(5):407–13.^10^ Creative Commons Attribution 4.0 International License (https://creativecommons.org/licenses/by/4.0/).

Chronic exposure to tobacco smoke leads to chronic inflammation and partial suppression of the innate immune system. When these factors are associated with genetic susceptibility, suboptimal lung maturation or chronic exposure to pathogens in some smokers, COPD may be more likely to develop (Fig. 3).^10^ Ageing and COPD share several common pathways and mechanisms, such as the accumulation of reactive oxygen species and senescent cells, or poor stem cell turnover, which is associated with a chronic inflammatory state and poor tissue repair.^11^ In addition, the immune system of COPD patients ages at a faster rate than healthy people of the same age.^12^

The different types of diffuse interstitial lung diseases have a significant relationship with immunological phenomena. Thus, it has been shown that there are senescence processes in the cellular pathways involved in pulmonary fibrosis. These mainly affect type 2 pneumocytes and pulmonary fibroblasts, which are the main drivers of lung repair and regeneration. Some triggers and molecular signalling pathways are shared between these cells, such as telomere shortening, autophagy, mitophagy, and the senescence-associated secretory phenotype (SASP) pathway, which causes a vicious circle by acting as both triggering and effector molecules.^13^

There is robust evidence of an increased risk of influenza infection and pneumonia in older patients. This is due to several causes, including impairment of the mucosal barrier (with reduced mucociliary clearance and immune response to micro-organisms), pulmonary inflammaging and a poorer response to vaccines.^8^, ^14^ In this regard, it has been shown that in immunosenescent patients there is a lack of immune response (both humoral and cellular) to antigens of various pneumococcal serotypes, whether acquired by infection or vaccination.^14^ In the case of COVID-19 there is also ample evidence of increased severity of infection in older patients. In addition to a greater number of comorbidities in this population, this may be due to an altered immune response, with a release of cytokines different from those found in younger patients.^15^

In summary, the change in global demographics as a consequence of the increase in life expectancy requires a greater clinical and research focus on the physiological process of aging and its impact on chronic diseases. Immunosenescence, influencing both innate and adaptive immunity, shapes the clinical “aging” phenotype seen in many chronic respiratory diseases, such as asthma, COPD, and pulmonary fibrosis, with an increased predisposition to respiratory infections. Vaccination, adequate sleep, proper nutrition and physical exercise can help prevent or reduce the consequences of immunosenescence and inflammaging.^16^

## Immune fitness

### What is immune fitness?

Immune fitness or immune training involves establishing lifestyle habits that can improve our immune capacity. We now know that having good eating habits, good social relationships, not smoking, limiting alcohol consumption, exercising, controlling stress levels and establishing a correct vaccination programme have a very positive impact on the quality of the immune response, even slowing down the process of immunosenescence.

### The link between immunity and nutrition

**Fig. 4:**
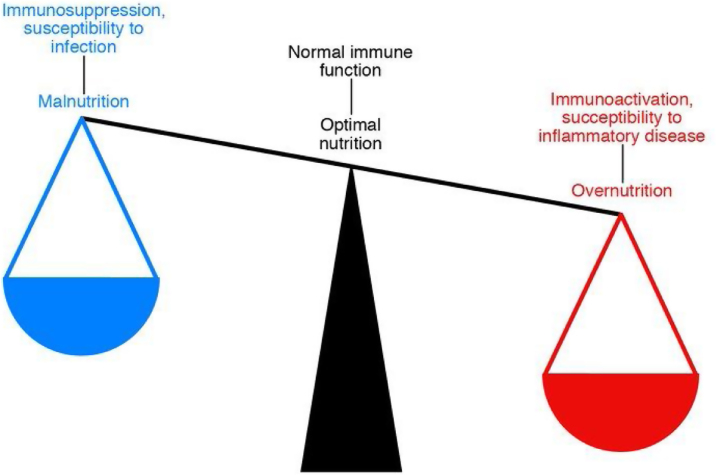
The delicate balance between nutrition and immunity.

Nutrition is closely related to the functioning of our immune system, and this relationship is bidirectional. This means that nutritional habits can modulate the immune response, making us more or less predisposed to suffer more or less severely from infectious and inflammatory diseases and even to develop some types of cancer (Fig. 4).^17^

Numerous studies have shown both the positive and negative effects of various nutrients on the immune capacity of individuals. Who has not heard of the anti-inflammatory effect of polyunsaturated fats, the immunostimulation exerted by various vitamins, trace elements and amino acids, or how obesity and ageing determine a state of “chronic inflammation” that is perfectly modifiable through diet? It is therefore clear that proper nutrition can help improve immune age.^16^

The Mediterranean diet or calorie restriction shows a very positive effect on the control of inflammation and oxidative stress, as well as establishing the conditions for maintaining a healthy and adequate microbiota. Other strategies are based on supplementation with micronutrients such as zinc and vitamins D, E and C, which have demonstrated their immunomodulatory capacity.^5^ Zinc is an essential micronutrient involved in the regulation of innate and adaptive immune responses. The main cause of zinc deficiency is malnutrition, leading to a state of immune dysfunction that prevents a proper response to infections. Zinc is an essential component of pathogen clearance signal transduction pathways that drive neutrophils to form extracellular traps (NETosis), as well as the induction of T- and B-cell-mediated immunity. In addition, zinc modulates the inflammatory response and controls both oxidative stress and the regulation of proinflammatory cytokines. Therefore, this micronutrient plays a significant role in immune function and homeostasis.^18^ Studies show that a significant percentage of older people show a decrease in zinc intake and absorption,^19^ which contributes to the process of immunosenescence.

The role of vitamin D in bone-calcium metabolism has been widely known for many years. However, its anti-infective and immunomodulatory effect is less well known. Vitamin D is the precursor of certain compounds known generically as “antimicrobial peptides”, including such rare ones as cathelicidins and defensins, which are also produced to a significant extent within the respiratory system.^20^, ^21^ Vitamin D is also capable of increasing the phagocytic capacity of neutrophils and macrophages. Apart from this anti-infective activity, one of the most interesting functions of this vitamin is its ability to regulate and control inflammation through the activation of regulatory T cells.^22^ It is not uncommon to find children and adults, and especially older adults, with vitamin D deficiency.

Moreover, several recently published studies have shown that the quality of the gut microbiota may be related to the response to infections and vaccinations.^23^ During aging, changes occur in the quality and biodiversity of the intestinal microbiota, significantly increasing the permeability of the intestine (Leaky Gut Syndrome), which may significantly contribute to the process of immune dysfunction. The relationship between microbiota, infection and vaccines has served as a starting point for research into whether the use of certain probiotics could improve responses to certain infectious agents and vaccines.

### Vaccination

The main objective of vaccination is the prevention of infection by specific pathogens. The direct impact of vaccination on the maintenance of homeostasis is achieved through the prevention of infection-induced complications. Other positive effects include enhancing the trained innate immune response by reprogramming innate cells (macrophages, dendritic cells, NK cells, etc.) to respond more efficiently to threats from other pathogens and slowing immunosenescence by altering the epigenetic clock and harnessing the pool of memory B and T cells to enhance responses to new infections. Vaccines may exploit the plasticity of the immune system to drive longer-term immune responses that promote health at a broader level than just the prevention of single, specific infections. Clearly, vaccines can positively contribute to “immune fitness” in ways and mechanisms that are only now beginning to be understood. Therefore, life-course vaccination is a fundamental tool for achieving healthy aging.^3^

### Exercise

Exercise has a significant effect on the regulation of the immune system. Exercise sessions induce an intensity-dependent effect. These processes are the result of the activation of the sympathetic nervous system and the hypothalamic-pituitary-adrenal axis, inducing the release of stress hormones such as catecholamines and glucocorticoids.

Regular exercise of moderate intensity exerts beneficial immunoregulatory effects on the control of inflammation associated with obesity, type 2 diabetes or cardiovascular diseases, also counteracting the effects of immunosenescence. Thus, physical exercise helps modulate the effects of aging, reducing the release of proinflammatory cytokines and senescent cells, and inducing the release of myokines (cytokines produced by muscle tissue) with anti-inflammatory properties.^24^ Older people who exercise daily show better levels of inflammation control, and better response to infections and vaccines. Consequently, exercise represents a powerful behavioural intervention that has the potential to improve both immune function and general health in subjects of all ages.

### Bio-psycho-social well-being

Numerous studies are now available that highlight the effect of stress and other psychological disorders on our defences. There is evidence that the way of coping with stress, mood and psychotherapy have a direct impact on the outcome and survival of patients with serious diseases such as AIDS, breast cancer or people at risk of cardiovascular disease (mainly myocardial infarction and stroke).^17^ In particular, a decrease in social activity and interaction with other people has a significant influence on the capacity of the immune system. Older people who live alone or who feel lonely often show a worse immune response to infection and even to vaccines.^25^

Because the mental state can affect immunity, certain psychological interventions can influence the course and outcome of diseases.

Another key factor that affects immunity is sleep, or rather the lack of it. Sleep deprivation is associated with immune dysregulation resulting in increased susceptibility to chronic inflammation, more common infections and poorer response to vaccines. On the contrary, adequate quantity and quality of sleep favour immune function, and reduce infectious risks, also improving responses to vaccination.^26^

### Drugs

Therapeutic strategies aimed at preventing or delaying the effects of senescence are currently being developed and are known as “senotherapies”. Due to space limitations and to summarise very briefly, senotherapy is based on two approaches; on the one hand, the use of senolytic products, which aim to eliminate the accumulation of aged or senescent cells largely responsible for all tissue ageing processes, and on the other, gerontoprotective treatments, which attempt to counteract the low-grade inflammatory conditions that develop during ageing.^27^ Senolytic strategies include molecules such as dasatinib, quercetin or the FOXO4 peptide, as well as treatments based on anti-senescent antibodies or CAR-T cell therapy.^28^, ^29^ Among the gerontoprotective drugs we have some well-known ones such as metformin, rapamycin or ruxolitinib.^29^, ^30^

In short, proper nutrition accompanied by good stress management and social relationships, along with routine exercise and the administration of appropriate, age-adapted vaccinations, can develop an optimal “immune pattern” on which overall health and immune status in particular depends. These measures, together with the future development of anti-ageing drugs and other vaccines such as the cytomegalovirus vaccine (which plays a key role in the immunosenescence process), will undoubtedly further improve our “immunobiography”, which will undoubtedly make a positive contribution to healthy ageing.

## Vaccination in the adult chronic respiratory patient

### Influenza

Respiratory infections caused by influenza viruses are highly prevalent and preventable. The indication for influenza vaccination is established in the adult vaccination schedule, has been key in reducing exacerbations of COPD and asthma, and forms part of the recommendations of most of its clinical management guidelines.^31^, ^32^, ^33^, ^34^ The efficacy of the influenza vaccine is approximately 60%, but its use results in a decrease in mortality and hospitalisation, especially in the over-65 population.^35^, ^36^ In the latter, seroprotection, measured as the immune response of haemagglutination inhibitor antibodies, decreases linearly from days 21–42 to 365 days.^37^

### Pneumococcus

Pneumococcal vaccination is the other well-established vaccine in the adult vaccination schedule and the various clinical guidelines for the management of respiratory diseases,^31^, ^32^, ^38^ as it has been shown to reduce exacerbations such as COPD.^39^ Its 13-serotype conjugate form (PCV13) provides adequate protection against invasive pneumococcal disease and its complications. The CAPiTA study found that the vaccine was 45.6% effective in preventing pneumococcal pneumonia caused by the vaccine serotypes and 45% effective for non-bacteremic pneumonia. It was also found to be remarkably effective (75%) in preventing invasive pneumococcal disease. On the other hand, it did not prove to prevent mortality due to pneumonia.^40^ Subsequent studies have shown greater and longer-lasting vaccine efficacy of PCV13 than 23-serotype polysaccharide (PPSV23) in immunocompetent subjects with risk factors for the vaccine serotypes in non-bacteremic community-acquired pneumonia, as well as in invasive pneumococcal disease.^41^, ^42^ On the other hand, and within the concept of virus-bacteria interaction, nasopharyngeal colonisation by pneumococcus could compromise the immune response against viruses such as influenza or SARS-CoV-2, so that the pneumococcal conjugate vaccine could have an added value in reducing the severity of these infections.^43^, ^44^

The new pneumococcal conjugate vaccines PCV15 and PCV20 have been approved for active immunisation for the prevention of invasive disease and pneumonia caused by *Streptococcus pneumoniae* in individuals 18 years of age and older. PCV15 provides protection against 15 serotypes of *Streptococcus pneumoniae* and PCV20 against 20.^45^, ^46^ The clinical development of both vaccines is based on the protection and safety demonstrated by PCV13. A recent study has shown that the new 20-serotype pneumococcal conjugate vaccine (PCV20) was safe and well tolerated, with an immunogenicity comparable to PCV13 and PPSV23. The immune response to PCV20 was noninferior to PCV13 and to 6 additional PPSV23 serotypes in participants aged ≥60 years. The PCV20 pneumococcal vaccine induced robust responses to all 20 vaccine serotypes in all age groups (≥60, 50–59, 18–49 years) and is expected to extend protection against pneumococcal disease in adults.^47^ PCV15 is well tolerated and induce immune response comparable to PCV13 for the 13 shared serotypes and elicits superior response for serotypes 3, 22F, and 33F.^48^ The fraction of disease attributable to the different pneumococcal vaccines in adults in Spain is shown in Table 1.^49^, ^50^ With the approval of these new conjugate vaccines, current vaccination recommendations should be revised, including the simplest schedule to implement, ensuring the best protection against adult pneumococcal disease.

**Table 1:** Disease fraction attributable to the different pneumococcal vaccines in adults in Spain.^49^, ^50^

	PCV13	PCV15	PCV20	PPSV23
All-cause CAP^a^	12.9%	14.5%	23.8%	NA
Pneumococcal CAP^a^	38.05%	42.7%	69.95%	NA
IPD^b^	25%	31%	62%	70%

CAP: Community Acquired Pneumonia, IPD: invasive pneumococcal disease, PCV13: 13-valent pneumococcal conjugate vaccine, PCV15: 15-valent pneumococcal conjugate vaccine, PCV20: 20-valent pneumococcal conjugate vaccine, PPSV23: 23-valent pneumococcal polysaccharide vaccine, NA: not applicable.

a Adults over 18, based on 2016–2018 data.

b Adults over 65, based on 2019 data.

### SARS-CoV-2 coronavirus

The SARS-CoV-2 coronavirus infection continues to cause millions of deaths worldwide and demonstrates the vulnerability of our immune system. Current vaccines for COVID-19 are not only safe and effective in preventing severe disease and mortality, but are currently the most useful preventive tool for pandemic control.^1^, ^51^ If SARS-CoV-2 infection can cause severe respiratory tract injury, especially in vulnerable patients with chronic respiratory disease, vaccination in these patients is a priority and has been reflected in the main clinical guidelines for the management of these diseases.^1^, ^31^, ^32^ The future of vaccination against SARS-CoV-2 will depend mainly on the effectiveness and nature of the vaccines, people's confidence in them and vaccine strategies together with the evolution of the virus in three different scenarios: the current pandemic, the early post-pandemic, where booster vaccination is likely to be carried out in the population susceptible to severe disease or in people who need it because of their susceptibility or social activity, and the late post-pandemic, where vaccination against SARS-CoV-2 for vulnerable people will depend more on the degree of population immunity rather than individual immunity. SARS-CoV-2 is likely to become endemic and require more specific attention in our adult vaccination schedules, especially in the chronic respiratory patient.^52^

### Bordetella pertussis

Pertussis is an infect

ious disease caused by *Bordetella pertussis*, highly contagious, underdiagnosed and with an increasing rate of infection and mortality in the adult population over 65 years of age, with recurrent flare-ups every 3–5 years, which neither natural immunity, which disappears at 4–20 years, nor vaccination in childhood, which disappears 4–12 years after the last dose, provides lifelong immunity.^53^ Therefore, the recommendation of pertussis vaccine in the adult vaccination schedule could go beyond a booster dose at 65 years of age or earlier, as its immunogenicity and safety profile is good and vaccination every 10 years with dTpa could be recommended. The Center for Disease Control and Prevention (CDC) already suggests this vaccination in adulthood every 10 years.^54^

Several studies have shown an increased risk of pertussis infection and hospitalisation in COPD patients. Additionally, having underlying COPD may contribute to the clinical severity of pertussis infections. This bidirectional relationship also appears to be present in asthma and its exacerbations.^55^ This has already been echoed in the most important COPD management guidelines, which already recommend vaccination with dTpa in adult COPD patients who were not vaccinated in adolescence.^31^, ^32^ In the future, and with the advent of more studies with robust results, asthma and COPD guidelines should include vaccination recommendations similar to those of the CDC.^55^

### Herpes zoster

Immunosenescence, immunocompromised states, and chronic respiratory diseases, such as COPD, increase the risk of herpes zoster (HZ) and postherpetic neuralgia. This risk increases particularly after the age of 50.^56^ Not only has a higher incidence of HZ been reported among COPD patients than among non-COPD patients, but COPD patients aged ≥ 50 years with HZ have a higher economic burden and higher burden of clinic and emergency department visits than those without HZ.^57^ Therefore, COPD patients and others with respiratory diseases aged ≥ 50 years or immunocompromised could benefit from HZ prevention through vaccination.

The efficacy, safety and duration of the new adjuvant recombinant HZ subunit vaccine (HZ/su) has been measured in several studies and shown to be safe, with an efficacy of 97.2% in individuals aged ≥ 50 years and 91.3% in those aged ≥ 70 years, which remains stable and high until 7.1 years after initial vaccination.^58^, ^59^, ^60^ In addition, efficacy against HZ has been observed to be around 84.5% in patients with respiratory disorders, such as asthma or COPD.^61^ In line with the CDC, the GOLD guidelines have included the recommendation of HZ vaccination in patients with COPD ≥ 50 years.^31^, ^62^ This vaccine should also form part of the vaccination schedule for respiratory patients.

### Respiratory syncytial virus

Human respiratory syncytial virus (RSV) is an important pathogen in paediatric populations, with reinfection occurring throughout life, with more severe disease in older adults, patients with cardiopulmonary disease and immunocompromised patients. It is also a global pathogen that will become increasingly important in developed nations with aging populations. At present, treatment is supportive. The development of effective antiviral agents for its treatment and vaccines for the prevention of RSV remains an important unmet medical need in the older adult population.^63^ Future vaccines will target the prevention of RSV disease in children, in children born to vaccinated mothers and in older adults.

### Adult vaccination schedule

With the previous data on the different vaccines, we could suggest a vaccination schedule in the adult chronic respiratory patient (Table 2), which would continue to include the annual influenza vaccine and the pneumococcal vaccines PPSV23, PCV13, PCV15 and PCV20 with indications similar to the current ones. It should also include dTpa pertussis vaccine every 10 years, and HZ vaccine in patients over the age of 50 (in immunocompromised patients from the age of 18). It remains to be seen how the COVID-19 pandemic will evolve in order to know whether SARS-CoV-2 infection will be endemic, and whether the indication for its vaccine will be annual, a booster in the face of the emergence of widely disseminated escape mutations, or whether it will be applied only to at-risk groups. RSV vaccine may be another vaccine to be added to the schedule, pending the results of its studies. Finally, other uncertainties will be the arrival or not of combination vaccines, such as influenza and SARS-CoV-2, and of universal influenza vaccines, or pan-coronavirus vaccines, to at least address all human betacoronaviruses.

**Table 2:** Vaccination schedule for adults: a perspective on the chronic respiratory patient.

Vaccine	Recommendation	Health agencies and authorities supporting the recommendation
Influenza	1 dose of influenza vaccine during the annual campaign	GOLD^31^, GeSEPOC^32^, GINA^33^, CDC^34^
Pneumococcus	1 dose of PCV followed by 1 dose of PPSV23, with a minimum interval of 8 weeks.	CDC^a^^,^^38^
	PCV13 and PPSV23 from the age of 65 and PPSV23 also recommended in younger chronic respiratory patients with other co-morbidities	GOLD^31^
	1 dose of PCV13	GesCOPD^32^
*Bordetella pertussis*	1 booster dose every 10 years with dTpa in adults who have not previously received a dose of this vaccine	GOLD^31^, CDC^53^
	Consider dTpa	GesCOPD^32^
Herpes zoster	2 doses of HZ/su starting at age 50	GOLD^31^, CDC^61^
SARS-CoV-2 coronavirus	Recommended depending on the evolution of the pandemic and the recommendations of the various governing bodies	

a Given the availability of 15- and 20-serotype pneumococcal conjugate vaccines in the United States, the current recommendation is to administer 1 dose of PCV15 or PCV20 to adult chronic respiratory patients. If PCV15 is administered, the recommendation is that a dose of PPSV23 should be administered at least one year later; whereas, if PCV20 is administered, subsequent vaccination with PPSV23 would not be recommended.

## Conclusions

Immunosenescence is the gradual deterioration of the immune system caused by advancing age. It is associated with a reduced ability to respond to infections and develop long-term immune memory. It plays a key role in the development of respiratory diseases that are more common in older people, such as asthma, COPD, diffuse interstitial disease and respiratory infections in the elderly.

We call immune fitness the establishment of lifestyle habits that can improve our immune capacity. We now know that good eating habits, good social relationships, not smoking, limiting alcohol consumption, exercising, controlling stress levels and establishing a proper vaccination programme can slow down the process of immunosenescence.

Influenza and pneumococcal vaccines (PCV13 and PPSV23 conjugate) are well established in the adult vaccination schedule. The new pneumococcal vaccines PCV15 and PCV20 will help to extend protection against pneumococcal disease in adults. The vaccine against COVID-19 is currently the most useful tool to prevent the disease and reduce its pathogenicity. It is highly recommended that vaccination against pertussis be carried out every 10 years. COPD patients and others with respiratory diseases aged ≥ 50 years or who are immunocompromised benefit from HZ prevention through vaccination. RSV vaccine may be another vaccine to be added to the schedule, pending the results of its studies. The arrival of combination vaccines, universal influenza vaccines or pancoronavirus vaccines are hopes that will become a reality in the coming years.

## Authors' contributions

All authors made substantial contribution to the design and drafting of the manuscript, and critical review of the content.

## Funding

The authors received no financial support for the authorship of this article.

## Conflict of interests

FV-Á declares having attended or participated in activities organised or funded by the pharmaceutical companies Almiral, AstraZeneca, Bial, Boehringer Ingelheim, Chiesi, GlaxoSmithKline, Esteve, Ferrer, Menarini, Novartis, Mundipharma, Orion, Pfizer, Teva and Zambon.

CAJ-R declares having attended or participated in activities organized or funded by the pharmaceutical companies Bial, Chiesi, Menarini and Pfizer.

DRC declares to have attended or participated in activities organized or funded by the pharmaceutical companies Boehringer Ingelheim, Chiesi, Gebro, Insmed, Novartis, Pfizer, Praxis, Teva and Zambon.

FF-G declares no conflict of interest.

FV-Á is part of the Editorial Panel of Open Respiratory Archives and declares to have remained outside the evaluation and decision-making process in relation to this article.
